# Accuracy of general hospital dementia diagnoses in England: Sensitivity, specificity, and predictors of diagnostic accuracy 2008–2016

**DOI:** 10.1016/j.jalz.2018.02.012

**Published:** 2018-07

**Authors:** Andrew Sommerlad, Gayan Perera, Archana Singh-Manoux, Glyn Lewis, Robert Stewart, Gill Livingston

**Affiliations:** a Division of Psychiatry, University College London, London, UK; b Camden and Islington NHS Foundation Trust, St. Pancras Hospital, London, UK; c Department of Psychological Medicine, Institute of Psychiatry, Psychology and Neuroscience, King's College London, London, UK; d INSERM U 1018, Epidemiology of Ageing and Age-Related Diseases, Villejuif, France; e Department of Epidemiology and Public Health, University College London, London, UK; f National Institute for Health Research Biomedical Research Centre, South London and the Maudsley NHS Foundation Trust, London, UK

**Keywords:** Diagnosis, Epidemiology, Prevalence, Medical records, Hospital records

## Abstract

**Introduction:**

Recognizing dementia in general hospitals allows for tailored care. We aimed to assess hospital dementia diagnosis accuracy, changes over time, and predictors of correct identification.

**Method:**

Retrospective cohort study of people over 65 years, using data from a large mental health care database as gold standard, linked to 2008–2016 English hospital data.

**Results:**

In 21,387 people who had 138,455 admissions, we found sensitivity and specificity of dementia recording, respectively, to be 78.0% and 92.0% for each person's complete records, and 63.3% and 96.6% for each nonelective admission. Diagnostic sensitivity increased between 2008 and 16. Accurate general hospital recording of the presence of dementia was lower in ethnic minority groups, younger, single people, and those with physical illness.

**Discussion:**

Dementia diagnosis recording in general hospitals is increasing but remains less likely in some groups. Clinicians should be aware of this inequity and have a higher index of clinical suspicion in these groups.

## Highlights

• Over three-quarters of people with dementia had it recorded at any time in their subsequent general hospital records.
• For each nonelective hospital admission, sensitivity was 63.3% and specificity was 96.6%.
• Diagnostic recording has increased between 2008 and 2016.
• Recording of dementia is less likely in people from minority ethnic groups and single people.

## 1 Introduction

There are increasing numbers of people with dementia [1], and they are more frequently admitted to general hospitals than those without dementia [2], due to their greater burden of physical and mental comorbidity, poorer nutritional status, and difficulties managing medication and seeking timely medical care. In the United Kingdom, around two-thirds of people with dementia are thought to have received a diagnosis [3], but this frequently comes late in the illness [4], which limits the provision of appropriate care and opportunities for patients to make future plans at an early stage. Increasing timely diagnosis is part of many countries' dementia strategy [5], and hospital admission may be an opportunity to improve dementia diagnosis.

Recognition of dementia in hospital inpatients is also important as hospital medical records should accurately reflect the person's clinical condition so that tailored inpatient care and discharge plans can be provided, particularly considering the effect of dementia on existing health conditions [6]. People with dementia may forget the contents of the agreed management plan, and lack of mental capacity means that people with dementia are often unable to make health care decisions [7], potentially requiring others to make decisions according to best interests principles [8].

Furthermore, understanding the accuracy of dementia diagnoses in general hospitals will also inform clinical research as routine medical records are an increasingly important method of case ascertainment for epidemiological studies. Hospital data in the United Kingdom have been used to address numerous research questions [9], [10], [11], but there are concerns about inaccurate or missed diagnoses [12], and previous studies found this to vary according to sociodemographic and clinical characteristics [13]. The sensitivity of dementia reporting in hospital discharge records has been estimated to be 70% in the United States [14], 51% in Finland [15], and between 26% and 43% in Swedish studies [16], [17], [18]. These studies have used cohort study assessments or clinical examinations as gold standard, but they have been relatively small, and none have examined data later than 2008 nor examined trends over time. Recent health policy to increase timely diagnosis [19] and greater health care professional awareness of the condition may have increased accuracy of subsequent diagnostic recording.

We sought to investigate the accuracy of recorded diagnoses of dementia in general hospitals in the United Kingdom, using data up to 2016. In particular, we aimed to

1. analyze the sensitivity and specificity of dementia diagnosis recording in general hospitals, using secondary mental health care data as gold-standard diagnostic status;
2. examine time trends in sensitivity and specificity of general hospital dementia diagnosis between 2006 and 2016; and
3. explore sociodemographic and clinical correlates of diagnostic accuracy.

## 2 Methods

### 2.1 Ethics statement

The Oxfordshire Research Ethics Committee C (reference 08/H0606/71 + 5) approved the data resources for secondary analysis.

### 2.2 Study setting and data source

We conducted a retrospective observational study using data from two linked data sets of routinely collected clinical data, described below in sections 2.2.1 and 2.2.2.

#### 2.2.1 The South London and Maudsley National Health Service Foundation Trust Biomedical Research Centre case register “Clinical Record Interactive Search” data extraction tool

The Clinical Record Interactive Search (CRIS) data resource provides pseudonymized electronic medical records from South London and Maudsley, one of Europe's largest secondary mental health care providers, which delivers a range of psychiatric care, including dementia assessment and management in memory clinics to a catchment area containing 1.2 million residents in four South London boroughs. Memory clinics are the primary dementia diagnostic service in the United Kingdom whose practice is to take referrals from other health and social care services (usually primary care) of people who have been identified as having possible dementia. There is no routine dementia screening in the United Kingdom.

In CRIS, pseudonymized data are extracted from structured fields in patients' electronic clinical records and from unstructured text within clinical records (including correspondence and case notes) with a natural language processing algorithm using General Architecture for Text Engineering software [20], which generates text strings associated with diagnostic statements. The accuracy of the General Architecture for Text Engineering software is described in detail in a previous publication [21]; it has been found to have precision of 0.99 for diagnosis. The CRIS data set has been used to examine a variety of dementia-related research questions [22], [23]. Data are available for all clinical records from January 1, 2006, and CRIS is linked to the Hospital Episode Statistics (HES) database, described in the following.

#### 2.2.2 National Health Service Digital HES

This data set contains clinical information about National Health Service care, collected directly by hospital providers and has been used in a number of research studies [24], [25], [26]. The data of interest for this study are records of general (nonpsychiatric) inpatient admissions to any hospital in England and the clinical diagnoses recorded on each hospital discharge summary by the treating clinical team. Diagnoses are recorded as International Statistical Classification of Diseases and Related Health Problems, 10th Revision [27] codes and each admission has up to 20 diagnostic codes. The method of admission (elective or nonelective) is also recorded [28]. Diagnoses recorded in HES are those clinically identified during the admission, obtained from correspondence with primary care, or derived from preexisting clinical records such as previous hospital medical records—some record systems prepopulate diagnosis fields with previously recorded chronic conditions. There was no routine practice of dementia assessment in English hospitals until 2012 when the U.K. Department of Health recommended case finding in older inpatients for possible dementia, by asking if any person who was admitted had a change in their memory lasting a year to the extent that it influenced functioning. This would be followed, if dementia was suspected, by referral to memory services [29], although we have no data reporting the extent of adoption of this practice.

### 2.3 Study participants

We retrieved records from CRIS (South London and Maudsley) of all patients aged 65 years or over who had been assessed (as part of ongoing follow-up or as first clinical contact) during the study window from January 1, 2008 to March 31, 2016. We did not include patients whose first electronic record of dementia was during 2006–2007 as we aimed to identify people with newly diagnosed dementia rather than those with a history of the condition. Those whose first CRIS recording was before 2008 would include many whose dementia was diagnosed before the inception of the data set and who were being followed up during 2006–2007. These data were linked to HES records over the same period. All mental health and dementia diagnoses in CRIS were extracted from structured fields in the electronic medical record where clinicians are required to record International Statistical Classification of Diseases and Related Health Problems, 10th Revision [27] codes or from unstructured text using the General Architecture for Text Engineering software, including dementia diagnosis (coded in CRIS as F00x-F03x). We retrieved the dates of, and diagnoses recorded for, each general hospital admission during the study window, including diagnosis of dementia (coded in HES as F00x-F03x, G30x, G31.0, or G31.8). We excluded those who had dementia in their CRIS records but were later diagnosed as having mild cognitive impairment (F06.7), as we judged this to mean these people had the dementia prodrome state [30] rather than clinical dementia.

### 2.4 Covariates

We extracted data from CRIS on participants' age, sex, ethnicity (white, Asian, black African/Caribbean, or other), marital status, and last recorded dementia subtype (Alzheimer's disease; vascular dementia; Lewy body dementias; other dementia [encompassing any other specified dementia type]; and unspecified dementia [where dementia etiology was not recorded]). We estimated the socioeconomic status from the 2010 Index of Multiple Deprivation, which is based on 37 indicators related to the patient's most recent address [31], with a higher score indicating more socioeconomic deprivation. Dementia severity was estimated from the most recently recorded Mini Mental State Examination (MMSE) [32] score at the time of hospital admission. Other aspects of clinical presentation were derived from CRIS using the Health of the Nation Outcome Scale (HoNOS), which is a standard instrument applied routinely in mental health care with adequate-to-good psychometric properties [33]. It comprises 12 subscale rating problems with agitation; self-injury; alcohol/drug use; cognition; physical illness; hallucinations; depressed mood; relationships; daily living function; living conditions; occupation or activities; other problems. Each domain is rated 0 (no problem) to 4 (severe or very severe problem). As ≥2 is seen to indicate a clinically significant problem, we dichotomized the HoNOS scores in each domain to facilitate interpretation: scores of 0 and 1 were grouped as no/minor problems and scores of ≥2 indicated problem in that domain. We did not use the cognitive subscale in our primary analyses due to its correlation with MMSE or the “other” subscale due to its nonspecific clinical meaning.

Age, sex, and ethnicity statuses were taken from the baseline recording, and other covariates were recorded at the time closest to the first hospital admission.

### 2.5 Analytic approach

We used the CRIS database record as the gold-standard definition of dementia because it includes records from the area's memory clinics, which are the principal U.K. dementia diagnostic services [19], [34] in which people are assessed by trained psychiatrists in consultation with the broader clinical team. Those not seen in memory clinics would usually have been assessed by psychiatrists in other secondary mental health care services. Included patients were all assessed as part of routine clinical practice. They had all received an International Statistical Classification of Diseases and Related Health Problems, 10th Revision diagnosis of dementia (therefore fulfilling standardized gold-standard criteria) or another mental disorder during the study window. Although formalized dementia screening assessment was not administered to all participants, dementia would have been considered as a differential diagnosis for people aged over 65 years with psychiatric disorder, and those with suspicion of dementia would have received standard diagnostic workup. We henceforth describe as “sensitivity” the proportion of people with dementia in CRIS who are correctly identified as having the condition in HES and as “specificity” the proportion of people without a dementia diagnosis in CRIS who are correctly identified as such in HES.

A single cohort would not be adequate to analyze sensitivity and specificity because CRIS and HES assessments rarely take place simultaneously, and for those with CRIS-diagnosed dementia, the date on onset is uncertain, and for those without such a diagnosis at their last CRIS assessment, we could not be certain that dementia did not develop later. Therefore, we analyzed people with and without a CRIS dementia diagnosis separately. To assess sensitivity, we examined all HES records after the CRIS dementia index date, which was the date of the first dementia diagnosis in the CRIS database and up to March 31, 2016. For specificity, we examined all HES records from January 1, 2008 and before the CRIS index date, which was the date of last assessment in the CRIS database for people without dementia. All statistical analyses were undertaken using STATA 14.2 (2017).

#### 2.5.1 Sensitivity of HES dementia diagnoses

We calculated the following:

1. Sensitivity of HES diagnosis for
  a. each patient (proportion of people with dementia who have dementia recorded in any subsequent HES records);
  b. each admission (proportion of admissions of a person with dementia, after their index date, which have dementia recorded in HES); and
  c. individual admission records for nonelective admissions only because some patients have multiple repeated admissions for very short elective procedures, for example, renal dialysis or chemotherapy, during which full diagnostic assessment is unlikely to have taken place.
2. Sensitivity of HES diagnosis for nonelective admissions within one year of diagnosis, stratified for year of admission, to evaluate time trends. We restricted this analysis to admissions within 1 year of CRIS dementia diagnosis as we aimed to ensure approximately equal dementia severity for each year in the study window. We judged that allowing a longer gap between CRIS and HES dementia assessment might bias findings due to ease of diagnosis of more severe dementia. We used chi-squared test to examine trend in sensitivity over time.
3. Sociodemographic and clinical predictors of the presence of dementia being correctly recorded in HES for each patient with dementia recorded in CRIS, using logistic regression. Univariate regression for each covariate and then multivariable analysis mutually adjusted for each covariate and for number of general hospital admissions.

#### 2.5.2 Specificity of HES dementia diagnoses

We calculated the following:

1. Specificity of HES diagnosis for:
  a. each patient (proportion of people without CRIS-diagnosed dementia for whom dementia is absent in all preceding HES records);
  b. each admission (proportion of admissions of a person without CRIS diagnosed dementia, before their index date, which have dementia absent in HES); and
  c. specificity of individual admission records for nonelective admissions only.
2. Specificity for each nonelective admission of people without dementia, stratified for year of admission, to evaluate time trends. We did not include admissions after March 2015 to ensure all study participants had at least one year of potential CRIS follow-up after hospital admission. Chi-squared test examined trend in sensitivity over time.
3. Sociodemographic and clinical predictors of the absence of dementia being correctly recorded in HES for each patient without CRIS-recorded dementia, using logistic regression. Univariate regression for each covariate and then multivariable analysis mutually adjusted for each covariate and for number of general hospital admissions.

#### 2.5.3 Additional analyses

Twenty-seven percent of people with dementia and 61% of people without dementia had missing data on at least one covariate. To avoid a loss of efficiency, we imputed missing covariate values using multiple imputation by chained equations [35]. Five imputed data sets were created using STATA's mi package by replacing missing values with simulated values from a set of imputation models using a model constructed from all potential covariates and outcome variables. We conducted logistic regression on each imputed data set and combined coefficients using Rubin's rules [36].

We conducted a post hoc sensitivity analysis using the cognitive subscale of HoNOS rather than MMSE because of a large amount of missing MMSE data for people without dementia.

## 3 Results

The study sample comprised 21,387 people. Of these, 8246 had dementia diagnosed in CRIS (South London and Maudsley) during the study period and 13,141 did not. The sociodemographic and clinical characteristics of the study sample and percentage of missing covariate data are summarized in Table 1. The mean age at dementia diagnosis was 82.2 years and 60.4% were female. For the people without dementia, mean age at index date was 77.9 years and 55.4% were female. The majority were from white ethnic background and African/Caribbean people formed the largest ethnic minority group. People in the sample were mostly married or widowed and Alzheimer's disease was the dementia subtype for around half of people with dementia and vascular dementia for a quarter. The median time between dementia diagnosis in CRIS and subsequent general hospital admission was 1.4 years (interquartile range 0.5, 2.7 years), and the time between CRIS assessment of people without dementia and prior general hospital assessment was 1.7 years (interquartile range 0.6, 3.5 years).

**Table 1:** Sociodemographic and clinical characteristics of participants

HES record	People with dementia n = 8246				People without dementia n = 13,141			
	Dementia diagnosed (n = 6429)		Dementia not diagnosed (n = 1817)		Dementia not diagnosed (n = 12,094)		Dementia diagnosed (n = 1047)	
	n	%	n	%	n	%	n	%
Age∗								
Mean (SD)	82.6 (6.8)		80.9 (7.4)		77.5 (8.2)		82.2 (7.8)	
65–69	272	9.0	163	4.2	2817	23.3	77	7.4
70–74	675	13.3	241	10.5	2390	19.8	122	11.7
75–79	1266	21.2	386	19.7	2342	19.4	200	19.1
80–84	1720	25.5	464	26.8	2069	17.1	244	23.3
85–89	1671	20.0	363	26.0	1534	12.7	233	22.3
90+	825	11.0	200	12.8	942	7.8	171	16.3
Missing	0		0		0		0	
Sex								
Female	3929	61.1	1053	58.0	6638	54.9	638	60.9
Missing	0		1		2		0	
Ethnicity								
White	5019	78.1	1273	70.1	9153	80.1	790	80.5
Asian	274	4.3	107	5.9	597	5.2	44	4.5
Black African/Caribbean	821	12.8	315	17.3	1232	10.8	108	11.0
Other	189	2.9	86	4.7	450	3.9	39	4.0
Missing	126		36		662		66	
Marital status†								
Married	2020	31.4	587	32.3	3620	33.5	253	27.1
Divorced	460	7.2	167	9.2	1260	11.6	78	8.4
Widowed	2580	40.1	612	33.7	3137	29.0	361	38.7
Single	1053	16.4	338	18.6	2804	25.9	242	25.9
Missing	316		113		1273		113	
Mean deprivation score (SD)†	27.2 (11.2)		27.8 (11.2)		26.8 (11.7)		27.5 (11.4)	
Missing	0		0		0		0	
Mean MMSE (SD)†	18.2 (6.2)		20.2 (5.9)		24.2 (5.5)		20.4 (6.8)	
Missing	870		269		6436		490	
Problem with (from HoNOS subscale)†								
Agitation	1321	20.6	222	12.2	1493	16.6	205	26.2
Self-injury	78	1.2	23	1.3	655	7.3	29	3.7
Alcohol/drugs	150	2.3	62	3.4	574	6.4	31	4.0
Cognition	5647	87.8	1282	70.6	2503	27.9	444	57.8
Physical illness	3895	60.6	1109	61.0	6253	69.4	613	78.5
Hallucinations	787	12.2	187	10.3	1504	16.8	171	22.3
Depressed mood	731	11.4	248	13.7	3408	37.9	252	32.6
Relationships	1064	16.6	257	14.1	1910	21.3	190	24.5
Daily living	4390	68.3	1020	56.1	4413	49.3	5391	70.1
Living conditions	733	11.4	226	12.4	1037	11.8	127	16.9
Occupation/activities	2141	33.3	505	27.8	2553	28.9	273	36.4
Missing‡	294		106		3325		297	
Last recorded dementia diagnosis								
Alzheimer's disease	3373	52.5	796	43.8				
Vascular dementia	1461	22.7	390	21.5				
Lewy body	201	3.1	54	3.0				
Other dementia	443	6.9	133	7.3				
Unspecified	951	14.8	444	24.4				
Median number of hospital admissions (IQR)	4 (2,6)		2 (1,3)		4 (2,8)		6 (3,11)	

Abbreviations: HES, Hospital Episode Statistics; HoNOS, health of the nation outcome scale; IQR, interquartile range; SD, standard deviation; MMSE, Mini Mental State Examination.

∗ For people with dementia, age is at time of first dementia diagnosis; for people without dementia, age is at time of last assessment.

† Characteristic nearest to first hospital admission.

‡ Figure for missing HoNOS score is for the HoNOS domain with most missing information.

### 3.1 Sensitivity of general hospital diagnoses of dementia

Of the 8246 people with dementia who were admitted to hospital, 6429 (sensitivity = 78.0%, 95% confidence interval 77.1, 78.9) had dementia diagnosis at any time in their general hospital records (Table 2). The 8246 people had 37,329 total admissions following their dementia diagnosis during the study period, and the proportion of the individual hospital records that included dementia was 50.3% (49.8, 50.8). Sensitivity for 26,894 nonelective hospital admission records was 63.3% (62.7, 63.9).

**Table 2:** Sensitivity and specificity of general hospital diagnoses of dementia 2006–2016 for each individual patient and for each individual admission

Sensitivity/specificity assessment	Number of true positives/total with dementia Sensitivity (95% CI)	Number of true negatives/total without dementia Specificity (95% CI)
For each patient	6429 / 8246 78.0% (77.1, 78.9)	12,094 / 13,141 92.0% (91.6, 92.5)
For each admission	18,769 / 37,329 50.3% (49.8, 50.8)	99,302 / 101,126 98.2% (98.1, 98.3)
For each nonelective admission	17,023 / 26,894 63.3% (62.7, 63.9)∗	46,973 / 48,650 96.6% (96.4, 96.7)†

Abbreviation: CI, confidence interval.

∗ Excludes 10,435 elective admissions.

† Excludes 52,476 elective admissions.

**Fig. 1:**
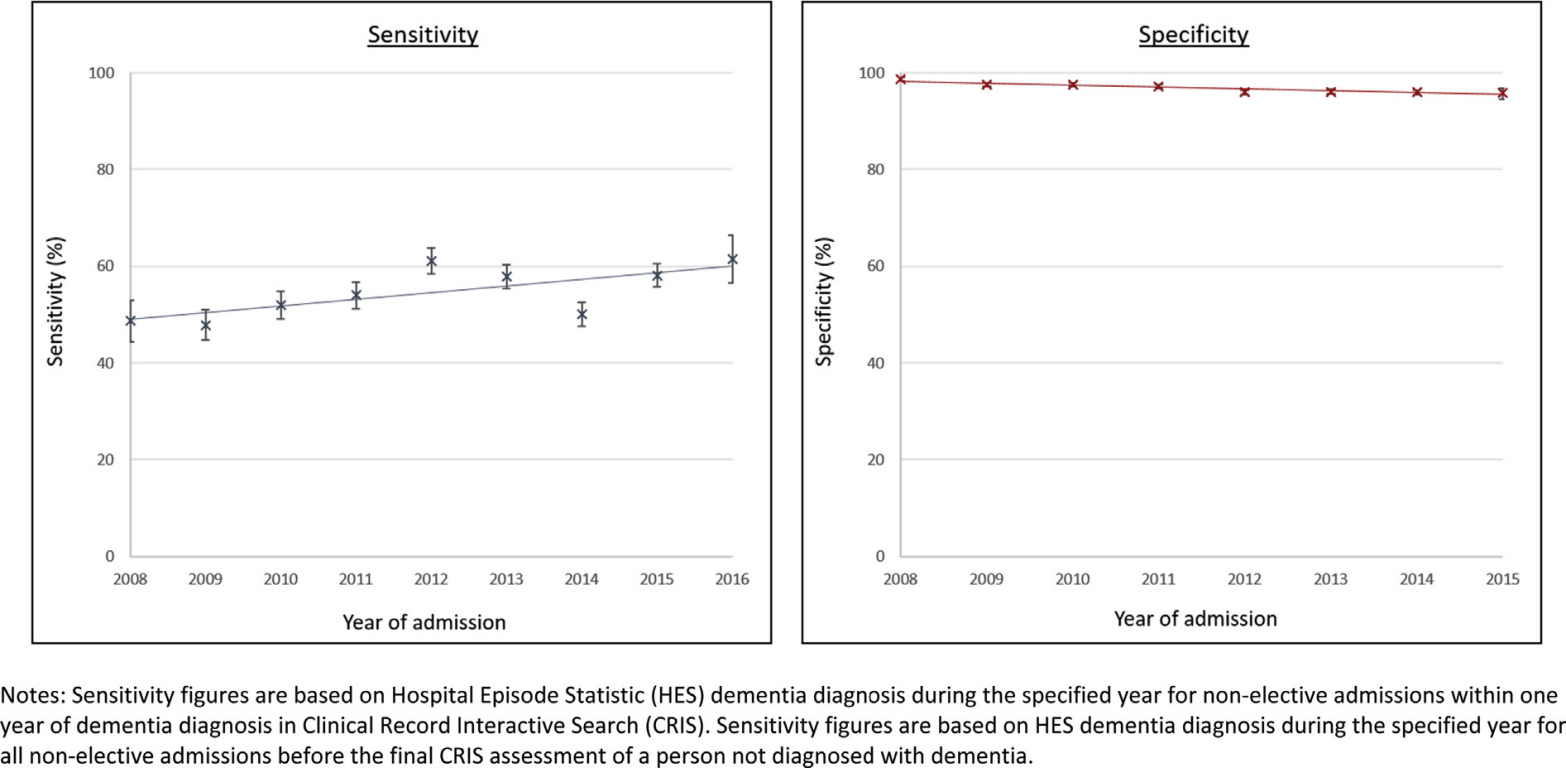
Sensitivity and specificity of general hospital dementia diagnoses during nonelective general hospital admissions between 2008 and 2016. Sensitivity figures are based on Hospital Episode Statistic (HES) dementia diagnosis during the specified year for nonelective admissions within 1 year of dementia diagnosis in Clinical Record Interactive Search (CRIS). Sensitivity figures are based on HES dementia diagnosis during the specified year for all nonelective admissions before the final CRIS assessment of a person not diagnosed with dementia.

Sensitivity of general hospital records within 1 year of CRIS diagnosis increased (P_trend_ < 0.001 [chi squared = 87.7, 8 df]) from 48.7% (95% confidence interval 44.3, 53.0) for admissions during 2008 to 61.5% (95% confidence interval 56.5, 66.4) for admissions in 2016 (Fig. 1, full data in Supplementary Appendix A.1).

In the fully adjusted multivariable model (Table 3), independent predictors of a person with dementia having it detected during subsequent general hospital admissions were increasing age, lower MMSE score, having previously recorded agitated behavior, problem with daily activities, and having more hospital admissions. People from nonwhite ethnic groups, single people, those with vascular or unspecified dementia, and those with problematic physical health were less likely to have a record of dementia in the HES database. Dementia recording increased with more hospital admissions. The fully adjusted model using a multiply imputed data set yielded similar results (Supplementary Appendix B).

**Table 3:** Predictors of the presence of dementia being correctly recorded in general hospital records of people with dementia: Univariate and multivariable logistic regression (n = 8246)

Characteristic	Univariate analysis		Mutually adjusted multivariable analysis (n = 6037)	
	Odds ratio	*P* value	Odds ratio	*P* value
Age (per 1 year increment)	1.03 (1.03, 1.04)	<.001	**1.04 (1.02, 1.04)**	**<.001**
Sex				
Female	1.14 (1.02, 1.27)	.01	1.00 (0.86, 1.17)	.97
Ethnicity				
White	1	<.001	1	**<.001**
Asian	0.65 (0.52, 0.82)		**0.61 (0.45, 0.83)**	
Black African/Caribbean	0.66 (0.57, 0.76)		**0.57 (0.47, 0.69)**	
Other	0.56 (0.43, 0.72)		**0.53 (0.37, 0.75)**	
Marital status				
Married	1	<.001	1	.17
Divorced	0.80 (0.66, 0.98)		0.89 (0.69, 1.14)	
Widowed	1.23 (1.08, 1.39)		0.98 (0.82, 1.17)	
Single	0.91 (0.78, 1.06)		**0.81 (0.67, 0.99)**	
Deprivation score (per 10-unit increase in deprivation)	0.95 (0.91, 1.00)	.04	0.94 (0.89, 1.01)	.08
MMSE (per 1-unit decrease)	1.09 (1.08, 1.10)	<.001	**1.10 (1.09, 1.11)**	**<.001**
Problem with (from HoNOS subscale)∗				
Agitation	1.84 (1.58, 2.15)	<.001	**1.65 (1.31, 2.07)**	**<.001**
Self-injury	0.95 (0.59, 1.51)	.82	0.72 (0.37, 1.40)	.33
Alcohol/drugs	0.67 (0.49, 0.90)	.008	0.79 (0.52, 1.20)	.27
Physical illness	0.95 (0.85, 1.06)	.34	**0.74 (0.63, 0.86)**	**<.001**
Hallucinations	1.20 (1.01, 1.42)	.04	1.12 (0.88, 1.42)	.35
Depressed mood	0.80 (0.68, 0.93)	.005	0.93 (0.75, 1.15)	.49
Relationships	1.19 (1.02, 1.37)	.03	0.95 (0.76, 1.18)	.64
Daily living	1.68 (1.50, 1.87)	<.001	**1.43 (1.22, 1.69)**	**<.001**
Living conditions	0.89 (0.76, 1.04)	.14	0.85 (0.68, 1.05)	.14
Occupation/activities	1.28 (1.14, 1.44)	<.001	1.10 (0.93, 1.29)	.28
Last recorded dementia diagnosis				
Alzheimer's disease	1	<.001	1	**<.001**
Vascular dementia	0.88 (0.77, 1.01)		**0.76 (0.63, 0.91)**	
Lewy body dementia	0.88 (0.64, 1.20)		0.99 (0.65, 1.50)	
Other dementia	0.79 (0.64, 0.97)		0.98 (0.74, 1.29)	
Unspecified dementia	0.51 (0.44, 0.58)		**0.41 (0.34, 0.50)**	
Number of admissions (per additional admission)	1.15 (1.13, 1.18)	<.001	**1.17 (1.14, 1.20)**	**<.001**

Abbreviations: HoNOS, Health of the nation outcome scales; MMSE, Mini Mental State Examination.

∗ HoNOS subscale, dichotomized to 0–1 (no or minor problem) and 2–4 (problem behavior); bold figures indicate *P* < .05 in multivariable analysis.

### 3.2 Specificity of general hospital records

Of the 13,141 people who did not have dementia diagnosed by CRIS (South London and Maudsley) and who were admitted to hospital before their last contact, 12,094 (specificity = 92.0% [91.6, 92.5]) did not have dementia entered at any time in their previous HES records (Table 2). These 13,141 people had 101,126 admissions before their last CRIS assessment, and the proportion of the individual HES records that did not include dementia was 98.2% (98.1, 98.3). Specificity in 48,650 nonelective hospital admission records was 96.6% (96.4, 96.7).

Specificity of HES dementia records has decreased (p_trend_ < 0.001 [chi squared = 117.0, 7 df]), with diagnostic specificity for admissions in 2006 being 98.7% (98.3, 99.0) and in 2015 being 95.8% (94.6, 96.8) (Fig. 1, full data in Supplementary Appendix A.2).

In the multivariable analysis (Table 4), dementia was more frequently entered on HES records of people without a CRIS dementia diagnosis if the person was older, had lower MMSE or problems with agitated behavior, activities of daily living or living conditions, and in those with more general hospital admissions. These identified predictors were also found in our sensitivity analyses accounting for missing data using multiple imputation (Supplementary Appendix B) or the cognitive HoNOS subscale rather than MMSE (Supplementary Appendix C).

**Table 4:** Predictors of the absence of dementia being correctly recorded in general hospital records of people without dementia: Univariate and multivariable logistic regression (n = 12,094)

Characteristic	Univariate analysis		Mutually adjusted multivariable analysis (n = 5187)	
	Odds ratio	*P* value	Odds ratio	*P* value
Age (per 1 year increment)	0.94 (0.93, 0.94)	<.001	**0.97 (0.95, 0.98)**	**<.001**
Sex				
Female	0.78 (0.69, 0.89)	<.001	0.93 (0.75, 1.15)	.49
Ethnicity				
White	1	.80	1	.97
Asian	1.17 (0.85, 1.60)		1.06 (0.65, 1.73)	
Black African/Caribbean	0.98 (0.80, 1.21)		0.94 (0.70, 1.28)	
Other	1.00 (0.71, 1.39)		1.04 (0.57, 1.88)	
Marital status				
Married	1	<.001	1	.80
Divorced	1.13 (0.87, 1.47)		0.99 (0.69, 1.43)	
Widowed	0.61 (0.51, 0.72)		0.95 (0.73, 1.25)	
Single	0.81 (0.67, 0.97)		0.87 (0.66, 1.16)	
Deprivation score (per 10-unit increase in deprivation)	0.95 (0.90, 1.00)	.06	1.01 (0.92, 1.10)	.90
MMSE (per 1 unit decrease)	0.91 (0.90, 0.92)	<.001	**0.92 (0.91, 0.93)**	**<.001**
Problem with (from HoNOS subscale)∗				
Agitation	0.56 (0.47, 0.66)	<.001	**0.70 (0.54, 0.91)**	**.008**
Self-injury	2.03 (1.39, 2.97)	<.001	1.64 (0.96, 2.79)	.07
Alcohol/drugs	1.64 (1.14, 2.28)	.008	1.50 (0.90, 2.50)	.12
Physical illness	0.62 (0.52, 0.74)	<.001	1.09 (0.85, 1.41)	.48
Hallucinations	0.70 (0.59, 0.84)	<.001	0.86 (0.66, 1.11)	.24
Depressed mood	1.26 (1.08, 1.48)	.003	1.01 (0.81, 1.26)	.92
Relationships	0.83 (0.70, 0.99)	.04	0.97 (0.75, 1.26)	.82
Daily living	0.41 (0.35, 0.49)	<.001	**0.66 (0.52, 0.84)**	**.001**
Living conditions	0.66 (0.54, 0.81)	<.001	**0.71 (0.54, 0.94)**	**.02**
Occupational function	0.71 (0.61, 0.83)	<.001	1.01 (0.81, 1.29)	.87
Number of admissions (per additional admission)	0.96 (0.95, 0.97)	<.001	**0.94 (0.93, 0.95)**	**<.001**

Abbreviations: HoNOS, Health of the nation outcome scales; MMSE, Mini Mental State Examination.

∗ HoNOS subscale, dichotomized to 0–1 (no or minor problem) and 2–4 (problem behavior); bold figures indicate *P* < .05 in multivariable analysis.

## 4 Discussion

In this study examining the accuracy of general hospital diagnoses of dementia, we report that overall sensitivity and specificity of hospital dementia diagnoses were 78.0% and 92.0% for each person's complete hospital records and 63.3% and 96.6% for each individual nonelective hospital admission. The rate of dementia diagnosis in HES is increasing but missed diagnosis is more likely in people who are from ethnic minority groups, single, younger people, and those with better cognitive function, less agitation, or activity of daily living impairment, and with more physical illness. Dementia being entered on HES records for a person who is subsequently assessed by secondary mental health care services without dementia being recorded is also becoming more common over time and is more likely with older age, worse cognitive function and problem with agitation, daily living activities, or living conditions. Having more hospital admissions was associated with higher rate of dementia recording.

Our sensitivity estimates are similar to other studies [14], [15], [16], [17], [18]. Previous studies have indicated milder dementia is less likely to be detected in data sources [37], [38], [39], [40], while being married [40], female, or living in a care home has also been found to increase the chance of dementia being diagnosed. Our study, in a more ethnically diverse population, adds that unrecorded dementia diagnosis in general hospitals is particularly likely for people from ethnic minority groups, who are around half as likely to have a record of dementia. For some, this may be because of impaired communication between them or their family and the assessing clinician [41]. As dementia awareness is lower in minority ethnic groups [42], patients and their families may be less likely to report the emergence of dementia, and clinicians may misattribute symptoms. Our findings suggest that further efforts are required by clinicians in general hospitals to identify dementia cases in people from minority ethnic groups, by reducing language barrier through use of interpreters, using culturally appropriate cognitive assessments, and potentially targeted case finding. The increased risk of missed diagnosis in the presence of physical illness suggests dementia may be misattributed to physical comorbidity. Our findings that unrecorded diagnosis is also more likely in younger people and those with better cognition and activities of daily living suggest that milder dementia is more often missed. The presence of agitation was probably associated with diagnostic recording as this symptom in an older person can be a trigger for thorough dementia assessment despite the absence of overt cognitive symptoms. We found that single people are less likely to have dementia detected, consistent with previous research findings [13], likely due to the absence of an informant's collateral history. Particular effort should be made to seek supporting information from informants in these groups, and inability to obtain such information should not preclude thorough diagnostic assessment.

Our novel finding of increasing general hospital recording of dementia is important, as recognition of dementia during hospital admissions allows the clinical team to make appropriate adjustments to their communication style, incorporate family members views on health care decisions, initiate specific treatment for dementia's symptoms and consider the effects of dementia on management of other comorbid conditions. The observed increase in recording probably reflects increasing health care professional awareness of dementia, increasing coding accuracy [22], [43] and greater communication between primary and secondary care. Furthermore, efforts in 2012 by the UK Department of Health to increase diagnosis rates in secondary care by case finding in older admitted people [29] may have also increased diagnosis in general hospitals, as supported by our finding of increased diagnostic sensitivity during that year.

Our specificity estimate of 92% was lower than figures of 98% [18] and 99% [16] from other studies. However, our analysis of specificity should be interpreted with caution, and the true figure may in fact be higher. Our analysis is based on a cohort of people in contact with secondary mental health care services who may be more likely than a general population to have symptoms resembling dementia. “False-positive” dementia diagnosis (i.e., diagnosis in HES when later assessment did not result in dementia diagnosis in CRIS) is a possible unintended consequence of the drive for earlier dementia diagnosis and potentially harmful. However, we found that older age, worse cognitive function, and problem with daily living activities and agitation predicted “false-positive” recording of dementia, and these are hallmarks of dementia, so some of these may actually represent correct diagnosis of dementia in the general hospital and incorrect diagnosis (i.e., failure to detect dementia) by CRIS, in which case the specificity is underestimated.

### 4.1 Strengths and limitations

This is the largest and most up-to-date analysis of hospital register dementia diagnoses, with sufficient data to allow the first analysis of changes in accuracy over time. We used a very large secondary care mental health register as gold standard against which to test accuracy of general hospital diagnosis, with natural language processing used to increase the accuracy of the CRIS register by picking up people whose diagnosis had been written in text records rather than in structured diagnosis fields.

Missed dementia in the CRIS record is possible, although it is based upon the assessment of trained psychiatrists from dementia services. We therefore restricted our sample to people aged over 65 years whom the mental health care service would have been likely to assess for dementia. As the CRIS data source is retrospective, we are not able to validate its accuracy by assessing participants, as used in other studies [44] as it would rely on information, in particular collateral history and cognitive examination, obtained for individual patients. Records are likely to be written in a way that reflects the clinician's overall clinical impression. Missed dementia diagnosis in CRIS may mean that sensitivity in this study is overestimated—we expect that people with dementia whose condition was missed in CRIS would also be more likely to have missed diagnosis in HES—and that specificity may be underestimated, as described previously.

National dementia recording rates are estimated to be around 72%, and estimates for people in this study's catchment area are similar (75%) [3], meaning that CRIS records will miss people with dementia because they have not presented to services. For individuals never seen in secondary mental health care services, therefore not in our CRIS cohort, HES diagnostic sensitivity may be worse as they may be more likely to have characteristics associated with lack of HES dementia recording. Finally, our sample was derived from a specific region in urban and suburban London, which could limit representativeness. However, this area has considerable ethnic and socioeconomic diversity, which allowed us to examine the effect of these factors on dementia recording, and the hospital records were from all of England, so our results are likely to reflect a range of hospital diagnostic practice.

### 4.2 Clinical implications and future research

UK efforts to increase dementia diagnosis rates in general hospitals have had success, but there is lower recording rates in some groups, likely due to communication difficulties, lack of an informant, or the presence of other causes of cognitive decline. It is therefore important that clinicians are aware of this inequity, and that they have a higher index of suspicion in these patient groups. Policymakers should consider more targeted case-finding approaches and providing training for hospital clinicians in dementia detection in these patient groups. Better sharing of diagnostic information between health care providers, such as automatic population of hospital databases with previously diagnosed conditions, would increase clinician awareness of comorbid conditions including dementia. Future prospective research should seek to identify in more detail the effect of factors such as native language, the presence of an informant, and physical comorbidities on dementia diagnostic accuracy.

Our study also clarifies the validity of hospital episode statistics as a tool for epidemiological and clinical research, and we found higher sensitivity than previous studies. We note the dynamic of increasing dementia recording over the past 9 years and that more hospital admissions improve diagnostic accuracy. However, using HES for case ascertainment may create systematic bias, especially with people from ethnic minorities, in whom dementia will be underestimated. These factors should be taken into consideration when researchers use these records.

**Research in Context**

1. Systematic review: We systematically searched the literature and found six studies examining general hospital record dementia diagnostic accuracy, using data from before 2008, with sensitivity between 28% and 70% and specificity between 94% and 99%.
2. Interpretation: Our study is the largest and most recent analysis. The sensitivity and specificity of general hospital dementia diagnoses was 78.0% and 92.0%, respectively, for each person's complete hospital records and 63.3% and 96.6% for individual hospital admissions. Dementia recording increased between 2008 and 2016. Dementia was more likely to be missed in minority ethnic, older, single people, and in those with milder dementia, non-Alzheimer's dementias, or physical illness.
3. Future directions: Clinicians should be aware of groups less likely to be accurately diagnosed. Future research should test whether training in diagnostic challenge in these groups can improve practice and reduce inequity. Future epidemiological studies using hospital dementia diagnoses should be aware of potential for systematic bias in these databases.

## Supplementary data

Supplemental Appendices A–C
