# Supplementary material for: Accuracy of general hospital dementia diagnoses in England: Sensitivity, specificity, and predictors of diagnostic accuracy 2008–2016
Source: Alzheimers Dement. 2018 Jul;14(7):933–43. doi: 10.1016/j.jalz.2018.02.012 (PMC6057268; doi:10.1016/j.jalz.2018.02.012)
Supplement: Supplemental Appendices A–C [file mmc1.docx]

Supporting information

**Appendix A.1: Sensitivity of admission-level general hospital records of dementia, stratified for the year of admission (2008-2016)**

| Year of hospital admission | Number of non-elective general hospital admissions for people with dementia, ≤1 year following diagnosis | Number of times dementia recorded in HES | **Sensitivity** | **(95% CI)** | |
| --- | --- | --- | --- | --- | --- |
| **2008** | 528 | 257 | **48.7** | **44.3,** | **53.0** |
| **2009** | 969 | 464 | **47.9** | **44.7,** | **51.1** |
| **2010** | 1161 | 604 | **52.0** | **49.1,** | **54.9** |
| **2011** | 1281 | 692 | **54.0** | **51.3,** | **56.8** |
| **2012** | 1430 | 874 | **61.1** | **58.5,** | **63.7** |
| **2013** | 1552 | 898 | **57.9** | **55.4,** | **60.3** |
| **2014** | 1539 | 771 | **50.1** | **47.6,** | **52.6** |
| **2015** | 1627 | 946 | **58.1** | **55.7,** | **60.6** |
| **2016** | 387 | 238 | **61.5** | **56.5,** | **66.4** |

Appendix A.2: Specificity of admission-level general hospital records of dementia, stratified for the year of admission (2008-2015)

| Year of hospital admission | Number of non-elective general hospital admissions of people without dementia, any time prior to last assessment | Number of times dementia absent from HES record | **Specificity** | **(95% CI)** | |
| --- | --- | --- | --- | --- | --- |
| **2008** | 4647 | 4585 | **98.7** | **98.3,** | **99.0** |
| **2009** | 6305 | 6149 | **97.5** | **97.1,** | **97.9** |
| **2010** | 6343 | 6184 | **97.5** | **97.1,** | **97.9** |
| **2011** | 6450 | 6272 | **97.2** | **96.8,** | **97.6** |
| **2012** | 6648 | 6384 | **96.0** | **95.5,** | **96.5** |
| **2013** | 6484 | 6232 | **96.1** | **95.6,** | **96.6** |
| **2014** | 5861 | 5628 | **96.0** | **95.5,** | **96.5** |
| **2015** | 1268 | 1215 | **95.8** | **94.6,** | **96.8** |

Notes for appendix 1: Sensitivity figures are based on Hospital Episode Statistic (HES) dementia diagnosis during the specified year for non-elective admissions within one year of Clinical Record Interactive Search (CRIS) dementia diagnosis. Sensitivity figures are based on HES dementia diagnosis during the specified year for all non-elective admissions before the final CRIS assessment of a person not diagnosed with dementia.

Key: CI = Confidence interval; HES = Hospital Episode Statistic

**Appendix B: Predictors of dementia correctly ever being recorded in general hospital records of people with dementia (true positives) and dementia correctly never being recorded in general hospital records of people without dementia (true negatives): multivariate logistic regression using multiple imputation**

| Characteristic | | True Positives | |  | True negatives | |  |
| --- | --- | --- | --- | --- | --- | --- | --- |
|  |  | **Odds Ratio** | **p-value** |  | **Odds Ratio** | **p-value** | |
| Age (per 1 year increment) | | **1.03 (1.02, 1.04)** | **< 0.001** |  | **0.94 (0.93, 0.95)** | **< 0.001** | |
| Sex | Female | 1.05 (0.93, 1.19) | 0.39 |  | **0.87 (0.75 1.00)** | **0.05** | |
| Ethnicity | White | 1 | |  | 1 |  | |
|  | Asian | **0.67 (0.52, 0.85)** | **0.001** |  | 0.98 (0.71, 1.36) | 0.92 | |
|  | Black African/Caribbean | **0.63 (0.54, 0.74)** | **< 0.001** |  | 1.02 (0.82, 1.28) | 0.84 | |
|  | Other | **0.63 (0.47, 0.83)** | **0.001** |  | 0.83 (0.57, 1.20) | 0.31 | |
| Marital status | Married | 1 | |  | 1 |  | |
|  | Divorced | 0.94 (0.76, 1.18) | 0.61 |  | 1.01 (0.77, 1.31) | 0.96 | |
|  | Widowed | 1.02 (0.88, 1.18) | 0.77 |  | 0.97 (0.81, 1.15) | 0.70 | |
|  | Single | **0.91 (0.76, 1.08)** | **0.28** |  | 0.95 (0.78, 1.15) | 0.61 | |
| Deprivation score (per 10-unit increase in deprivation) | | **0.95 (0.90, 1.00)** | **0.04** |  | 0.98 (0.92, 1.04) | 0.46 | |
| MMSE (per 1 unit decrease) | | **1.06 (1.05, 1.07)** | **< 0.001** |  | **0.95 (0.94, 0.96)** | **< 0.001** | |
| Problem with^a^: | Agitation | **1.68 (1.74, 2.00)** | **< 0.001** |  | **0.73 (0.61, 0.88)** | **0.001** | |
|  | Self-injury | 0.79 (0.47, 1.31) | 0.36 |  | **1.52 (1.04, 2.24)** | **0.03** | |
|  | Problem-drink/drugs | 0.74 (0.53, 1.04) | 0.09 |  | 1.20 (0.76, 1.92) | 0.42 | |
|  | Physical illness | **0.74 (0.65, 0.85)** | **<0.001** |  | 1.15 (0.96, 1.38) | 0.12 | |
|  | Hallucinations | 1.04 (0.86, 1.25) | 0.71 |  | 0.86 (0.72, 1.03) | 0.10 | |
|  | Depressed mood | **0.78 (0.65, 0.93)** | **0.005** |  | 1.15 (0.98, 1.36) | 0.08 | |
|  | Relationships | 0.95 (0.80, 1.12) | 0.52 |  | 0.97 (0.78, 1.19) | 0.74 | |
|  | Daily living | **1.46 (1.27, 1.68)** | **< 0.001** |  | **0.71 (0.58, 0.88)** | **0.002** | |
|  | Living conditions | 0.87 (0.73, 1.04) | 0.13 |  | **0.78 (0.62, 1.00)** | **0.05** | |
|  | Occupational function | 1.10 (0.96, 1.26) | 0.16 |  | 0.98 (0.83, 1.16) | 0.83 | |
| Last recorded dementia diagnosis | Alzheimer’s Disease | 1 | |  |  | | |
|  | Vascular dementia | **0.80 (0.69, 0.93)** | **0.003** |  |  |  |  |
|  | Lewy body dementia | 0.94 (0.67, 1.32) | 0.72 |  |  |  |  |
|  | Other dementia | 0.85 (0.68, 1.06) | 0.14 |  |  |  |  |
|  | Unspecified dementia | **0.45 (0.38 0.52)** | **< 0.001** |  |  |  |  |
| Number of admissions (per additional admission) | | **1.19 (1.17, 1.22)** | **< 0.001** |  | **0.95 (0.94, 0.96)** | **< 0.001** | |

Key: HoNOS = Health of the nation outcome scales; MMSE = Mini-mental state examination

Notes: ^a^ HoNOS subscale, dichotomised to 0-1 (no or minor problem) and 2-4 (problem behaviour); Bold figures indicate significant results in multivariate analysis (p<0.05)

Appendix C: Predictors of the absence of dementia being correctly recorded in general hospital records of people without dementia: multivariable logistic regression using cognitive subscale of HoNOS rather than MMSE (n=8,528)

| Characteristic | | Mutually adjusted multivariable analysis | |
| --- | --- | --- | --- |
|  |  | **Odds Ratio** | **p-value** |
| Age (per 1 year increment) | | **0.95 (0.94, 0.96)** | **< 0.001** |
| Sex | Female | **0.79 (0.66, 0.95)** | **0.01** |
| Ethnicity | White | 1 | 0.34 |
|  | Asian | 0.80 (0.55, 1.17) |  |
|  | Black African / Caribbean | 0.82 (0.63, 1.06) |  |
|  | Other | 1.01 (0.61, 1.67) |  |
| Marital status | Married | 1 | 0.62 |
|  | Divorced | 0.91 (0.67, 1.25) |  |
|  | Widowed | 0.87 (0.70, 1.09) |  |
|  | Single | 0.87 (0.69, 1.11) |  |
| Deprivation score (per 10-unit increase in deprivation) | | 0.96 (0.89, 1.03) | 0.26 |
| Problem with  (from HoNOS subscale)^a^: | Agitation | **0.71 (0.57, 0.89)** | **0.002** |
|  | Self-injury | 1.46 (0.96, 2.23) | 0.08 |
|  | Alcohol/drugs | 1.25 (0.82, 1.90) | 0.29 |
|  | Cognition | **0.36 (0.30, 0.42)** | **<0.001** |
|  | Physical illness | 1.15 (0.92, 1.43) | 0.23 |
|  | Hallucinations | 0.87 (0.70, 1.08) | 0.21 |
|  | Depressed mood | 1.08 (0.90, 1.30) | 0.40 |
|  | Relationships | 1.02 (0.82, 1.26) | 0.89 |
|  | Daily living | **0.68 (0.56, 0.84)** | **< 0.001** |
|  | Living conditions | **0.66 (0.52, 0.84)** | **0.001** |
|  | Occupational function | 1.01 (0.84, 1.22) | 0.92 |
| Number of admissions (per additional admission) | | **0.94 (0.93, 0.95)** | **< 0.001** |
